# Cystic Fibrosis Treatment: A Paradigm for New Pediatric Medicines, Globalization of Drug Development and the Role of the European Medicines Agency

**DOI:** 10.3390/children2010108

**Published:** 2015-03-23

**Authors:** Klaus Rose, Michael G. Spigarelli

**Affiliations:** 1 klausrose Consulting, Aeussere Baselstrasse 308, 4125 Riehen, Switzerland; 2 Internal Medicine and Pharmacy, Division of Clinical Pharmacology, University of Utah, Salt Lake, UT 84108, USA; E-Mail: michael.spigarelli@hsc.utah.edu; 3 Divisions of Adolescent Medicine and Clinical Pharmacology, Clinical Trials Office, University of Utah, Salt Lake City, UT 84108, USA

**Keywords:** better medicines for children, pediatric drug development, cystic fibrosis, pediatric legislation, pediatric pharmaceutical legislation, EU pediatric regulation, FDA Innovation and Safety Act (FDASIA), Pediatric Committee (PDCO)

## Abstract

The European Pediatric Pharmaceutical Legislation wants children to benefit more from pharmaceutical progress. In rare diseases, concerns have been raised that this legislation might damage research and stymie drug development. We discuss the role of the European Medicines Agency (EMA) and its Pediatric Committee (PDCO) in the development of ivacaftor, first-in-class for cystic fibrosis (CF) patients with the G551D mutation (and eight other mutations later) and of lumacaftor and ataluren, two more potential break-through CF medications. Ivacaftor was USA-approved early 2012 and six months later in the EU. Registration was based on the same data. We analyzed these drugs’ EU pediatric investigation plans (PIPs) and compared the PIP-studies with the pediatric CF studies listed in www.clinicaltrials.gov. The ivacaftor PIP studies appear to reflect what the developer planned anyway, apart from a study in 1–23-month-olds, which has not yet started. The total negotiation time for the current PIP version was approximately 5.5 years. For companies that develop drugs in pediatric diseases, e.g., CF, PIPs represent considerable additional procedural workload with minimal or no additional benefit for the patients. New drugs for pediatric diseases should not be hampered by additional, unnecessary and costly bureaucracy, but be registered as rapidly as possible without compromising safety.

## 1. Cystic Fibrosis (CF)

Cystic fibrosis (CF) was first described in 1938 [1], but its symptoms were known to observant medical practitioners throughout the ages. Life expectancy was historically a year or less, and this remained unchanged until well into the 20th century. While CF patients had been historically treated only by pediatricians and pediatric subspecialists, they now routinely reach adulthood and transition into care by adult providers.

The fundamental physiologic issue in CF patients is that they cannot produce normal sweat, digestive fluids or mucus due to a defective transport protein, the cystic fibrosis transmembrane conductance regulator (CFTR). The basis for this deficiency is a mutation within the gene that codes for the CFTR protein. Approximately 1500 different mutations have been described. These different mutations result in lack of normal viscosity within these various fluid secretions that cumulatively damage the digestive tract, leading to repetitive bacterial lung infection and inflammation cycles and damaging most other organ systems. Children with CF suffer from delays in development, impaired weight gain and chronic organ damage by repetitive non-bacterial and bacterial infections. All told, this results in a life expectancy that currently averages 40 years [2,3,4].

## 2. Treatment of CF

Treatment of CF is multidisciplinary, involving drug therapy, ergotherapy, nutritional support, physical and psychological support of children and parents and a variety of other interventions. Drug therapy and physical therapy focus on specific organs, e.g., lungs, digestive system, reproductive system and others. A new paradigm is evolving: treatment of the disease to prevent the creation of damaging thick mucus, *i.e*., finding ways to help the body to produce normal viscosity mucus and secretory fluids [2,3,4].

Ivacaftor is the first of these new therapies. It was specifically designed to help CF patients with the G551D CFTR mutation; 4%–5% of all CF patients have this mutation [5,6]. Essentially, ivacaftor corrects the sweat and fluid production of these patients and has the potential to allow an almost normal life, as long as the medication is taken. Two pivotal 48-week, placebo-controlled clinical studies involving 213 patients, one in patients 12 years and older and another in patients between six and 11 years, demonstrated safety and efficacy in CF patients with the G551D mutation. In both studies, the primary efficacy endpoint was improvement in lung function (mean absolute change from baseline in percent-predicted predose the forced expiratory exhaled lung volume within one second (FEV_1_)) through 24 weeks of treatment. Ivacaftor demonstrated a statistically-significant improvement compared to placebo at Week 24 with a 10.6% improvement in adult and adolescent patients and a 12.5% improvement in children six to 11 years old; these improvements were maintained through Week 48 in both studies. The key secondary endpoints included: change in weight, time-to-exacerbation and patient-reported respiratory symptoms, such as cough, wheezing, *etc.* Each of these secondary endpoints supported the primary efficacy endpoint, as did the pharmacodynamic endpoint of reduction in sweat chloride concentrations. The FEV_1_ improvement measured in these two pivotal trials was demonstrated, in conjunction with standard-of-care-therapies and respiratory treatments recommended for patients with cystic fibrosis, with the exception of the use of hypertonic saline. The improvement in patient weight was demonstrated for ivacaftor in addition to standard of care pancreatic enzyme replacement therapy, vitamin and mineral supplementation, GI medications and additional dietary management, including increased caloric intake and additional supplementation with fat/carbohydrates/proteins. The improvements in both lung function and weight were regarded by the FDA as robust evidence of efficacy in the subpopulation of patients with CF with at least one copy of the G551D mutation within the CFTR [7,8,9,10,11]. The two pivotal studies were preceded by a clinical dose finding study and complemented by two further studies: one study similar in design to the two mentioned pivotal studies, but in CF patients homozygous for the F508del mutation (the most prevalent mutation) in the CFTR gene and a follow-up safety study, into which most study participants of all ivacaftor studies where switched.

Ivacaftor was registered by the European Medicines Agency (EMA) in July, 2012, on the basis of the same pivotal and additional trials that formed the basis of the FDA labeling decision. Detailed documentation of this decision is on the EMA website [12].

## 3. Modern Drugs, Drug Labels and Children

The industrial revolution allowed the production of chemicals on a large scale and, eventually, the use of some of them as drugs. Modern medicines are like a sword that cuts both ways: their therapeutic potential is often enormous, e.g., antibiotics or insulin, while they also have the potential for disastrous consequences. Disasters have molded pharmaceutical legislation. The thalidomide catastrophe of 1962 led to the Kefauver-Harris amendments in the U.S., mandating drug manufacturers to perform adequate clinical trials to demonstrate the safety and efficacy of the respective drug. Since that time, modern drug labels are only allowed to claim what has been proven in clinical trials. In reaction to this legislation, pharmaceutical manufacturers introduced pediatric disclaimers, which stated that the respective drug had not been tested in children. This was done to limit the legal liability if something went wrong. As a result, the vast majority of medicines used in the treatment of children have been mostly prescribed in an off-label manner [13,14,15].

With the increasing availability of modern drugs, clinical pharmacology evolved, investigating the absorption, distribution, metabolism and excretion (ADME) of drugs [16]. This led to the emergence of pediatric clinical pharmacology. In younger children, the organs are not yet mature, and the liver, kidney and other organs function different from adults. Key lessons were summarized by Kearns in 2003: dosing in children cannot be simply deduced from adult doses in relation to children’s body weight or body surface. This difference is more pronounced in smaller and younger children, as organ systems do not mature in parallel; and the variability of ADME in children is much higher than in adults [17]; thus demonstrating the old adage that children are not small adults.

## 4. Pediatric Pharmaceutical Legislation U.S. and EU

U.S. pharmaceutical pediatric legislation was introduced with the FDAMA (Food and Drug Administration Modernization Act) in 1997 and reauthorized several times, until it became permanent in 2012 within the FDA Safety and Innovation Act (FDASIA) [15]. It provides voluntary rewards to pharmaceutical companies in exchange for pediatric studies. Its key feature is the intention to provide clinicians with additional information on pediatric ADME (absorption, distribution, metabolization, excretion) and, consequently, on dosing and/or contraindication of patent-protected medicines to improve the care of children. The introduction of U.S. pediatric legislation reflected and was made possible by the progress made in pediatric clinical pharmacology. Pediatric clinical pharmacologists were part of the main driving force that led to the U.S. pediatric legislation. This was later complemented by a mandatory legislation, which gives FDA the authority to mandate pediatric studies (Pediatric Research Equity Act, PREA). PREA is restricted to the treatment of the same disease as in adults; rare and orphan diseases are excluded from PREA requirements [18]. The U.S. pediatric community welcomed the U.S. pediatric legislation. As CF is an orphan disease, it is exempt from PREA requirements. Companies that develop drugs for the treatment of orphan diseases can negotiate with the FDA for the pediatric exclusivity of a six-month patent extension in return for additional pediatric studies in another indication.

The EU pediatric legislation came into force in 2007. Triggered by the U.S. legislation, the desire was to go further and to achieve more tangible results for the treatment of children. In the U.S., pediatric discussion with the FDA did not occur before a new drug was granted marketing approval for adults. In the EU, this was designed to be different. Before a company can register a new drug in either adults or children, it needs a pediatric investigation plan (PIP) approved by the “Pediatric Committee” (PDCO), which is part of the European Medicines Agency (EMA). Without such an approved PIP, the registration process for the new drug is blocked [15,19,20,21].

Within the EU, the EMA PDCO does not grant drug approval. The decision regarding approval of a new drug is done by another committee, the CHMP (Committee on Human Medicine Products). As is routine for any other drug, as well, the decision to approve ivacaftor was made by the CHMP. A limited number of PDCO members are also CHMP members, but the two committees retain their separate roles and function. Without a successful negotiation and a positive PIP opinion, the dossier for approval by the CHMP cannot be submitted.

The EMA PDCO is composed of 66 members and alternates, most of them representing the now 28 EU Member States plus a few more associated countries (e.g., Iceland), with representation from the above-mentioned CHMP, patient advocacy groups and healthcare professionals. When the PDCO votes, only one of the two (member/alternate) may vote; but companies are required to send PIP information electronically and physically as CD-ROMs to all PDCO members. Within the EMA, a department of approximately 30 people runs the administrative portion of the PIP [15].

It goes without saying that the introduction of new drugs is essential for both patient treatment and the research-based pharmaceutical industry. Any unnecessary delay in the approval of new drugs can be potentially deadly for a patient and detrimental for a company. The concern for delays can affect decisions to move forward with new chemical entities that could benefit numerous children and their families.

The PIP, by regulatory mandate, must be submitted at the end of main human pharmacokinetic studies, *i.e*., before the efficacy of the drug in humans could be known. All drugs, including those drugs targeting rare and ultra-rare diseases, are required to have a PIP, even for those diseases that are exclusively found in children. The EU legislation expects a full pediatric registration. The PIP is intended to cover all potential future pediatric uses of the new drug as deemed possible by PDCO. In the U.S., mandatory pediatric trials are only required to cover the same indication as adults. The EU interprets the indication that a company is targeting as part of a “condition” and assumes that whatever pediatric disease can be defined within this condition, it must be addressed within the PIP [22]. As a consequence, a drug company developing a medicine against postmenopausal osteoporosis must justify its focus on this disease. If the justification is not accepted, EMA/PDCO requests (“asks”) the company to develop the drug for another type of osteoporosis that exists in children, e.g., steroid-induced osteoporosis [23]. The company must “propose” pediatric investigation “measures”, which can range from technical formulations (e.g., pills *vs.* liquids), juvenile animal trials, clinical trials in children, and others. If a company's proposal is regarded as not sufficient, the PDCO will issue a negative opinion and thereby block drug registration [15,18,19,20,21,23].

The PIP procedure takes three months in preparation prior to the start of the core procedure, seven months during the core procedure (an initial block of 120 days, a clock stop of usually three months (but can be much longer), a second block of 120 days and one month until the final PDCO decision becomes official). At the end of that time, PDCO issues a positive or negative opinion. In the event of a negative opinion, which would block registration, the company can either ask for a re-examination (by PDCO), start a new PIP procedure or stop development of the drug. If none of these options fit, the company can also sue at the European Court of Justice. Only one company has sued thus far and has lost in two instances [24,25]. This company is no longer in existence. As PIPs must be submitted early in the development process, a process is available for PIP modification requests. These take three months of preparation time and then only the first 120 day block of the PIP procedure, plus the usual post-PIP month, which results in a total process time of six months. The EMA estimates the number of PIP modifications necessary during a PIP life cycle to be three to five [26].

## 5. Development and Registration of Drugs in Cystic Fibrosis

Some elements of drug development are the same for all diseases in all age groups. A new chemical or biological entity, *i.e*., a molecule, an antibody or a combination thereof, will be investigated first for basic safety and carcinogenicity in the laboratory, later in animals and then in healthy adult volunteers, unless the new compound is so toxic, that testing is only ethically justifiable in a malignant or life-threatening disease, where the potential benefit outweighs the potential risk. At a later stage, efficacy data are collected in one or several “proof-of-concept” studies initially. The key studies that will allow a registration of a new compound are usually two “pivotal” phase 3 studies: the clinical endpoints that measure the efficacy of the new compound have to be predefined, and the compound will have to be measured against either a comparator drug or against placebo, *i.e*., no drug at all. To prevent psychological effects, neither the patient nor the treating physician knows which patient gets which drug (double-blind treatment) [27,28].

In CF, a well-recognized clinical endpoint for clinical studies is FEV_1_, *i.e*., the forced expiratory exhaled lung volume within one second. This endpoint works well for patients over six years old. The regulatory authorities face several challenges for primary and secondary endpoints to accept in clinical studies: the generally improved medical care of a CF patient. With an improved FEV_1_ value due to, e.g., physiotherapy, an additional improvement attributed to a new medication is not easy to prove [9,29].

The discussion about acceptable endpoints brings two distinct worlds together. Regulatory authorities who authorize the sale of medications in their respective territory have a legitimate stake in the discussion. They have an intrinsic duty to protect the patients against wrong or exaggerated claims. Life threatening diseases where therapeutic options are limited attract a lot of businesses that hope to exploit the hope and despair of patients and their relatives. Magnetic healing, witchcraft, shamanistic blessings and many other therapeutic principles are offered today in advertisements and on the Internet. However, only authorized medicines that have proven their efficacy are allowed to be prescribed by physicians and sold in pharmacies. Drug development is increasingly international, complex and expensive [30,31,32,33]. The key competence of regulatory authorities is the critical examination of claims submitted by pharmaceutical companies. By the nature of their business, companies are tempted to interpret their clinical studies in a way that is favorable towards their interests, and hence, regulatory authorities have to examine submitted data carefully. The clearer a protocol defines clinical endpoints and the interpretation of future results, the less controversy there will be in the regulatory interpretation of the results. Additional competencies by regulatory authorities lie in proactive discussion with drug developers about the number of clinical trials needed for registration, usually a standard preclinical (*i.e*., laboratory and animal) testing program, phase 1 studies in healthy volunteers, phase 2 proof-of-concept trial(s) and, as the key element, usually two pivotal phase 3 efficacy trials. For orphan designations, *i.e*., rare diseases where the number of necessary patients for two classically-designed pivotal phase 3 trials do not exist or would require too expensive logistics, health authorities can modify their requests on the proof of safety and efficacy [27,28]. Of course, these are case-by-case decisions taking into consideration the available medications, the benefit-to-risk ratio and many additional factors. Both FDA and EMA also offer scientific advice to drug developers. To some degree, regulatory authorities have a margin of discretion in these difficult and delicate situations [34,35]. The current challenges in defining endpoints in CF were discussed at an EMA-sponsored workshop in 2012 [29].

FEV_1_ was the clinical endpoint in the two pivotal studies that led to the registration of ivacaftor in 2012 by the FDA. A tough question is how to assess patients younger than six years of age, as they cannot routinely perform an FEV_1_ measurement. FEV_1_ measurement is possible in patients younger than six years, but only in specialized centers, which makes the trial logistics much more complicated. The challenge becomes more difficult the younger the patients to be tested, and FEV_1_ in patients < 2 years is definitely impossible, so pharmaceutical developers and regulatory authorities discuss other measurement types, e.g., computer-assisted measurements of thorax extensions and others [29].

## 6. PIPs for Pediatric Drugs

In contrast to the U.S., the EU legislation asks for a PIP for every single new drug, irrespective of whether it aims at an adult or a child disease. Obviously, it makes sense that a regulatory agency asks for a child-friendly formulation in a drug that a manufacturer plans to market only in adults and, hence, plans to register only tablets, which cannot be swallowed by children < 6 years of age, in the likelihood that the drug will be used in children. This is in contrast to what happens with drugs that are developed for children. A company that develops a drug for a pediatric disease must know and understand pediatric development and physiology and the disease in depth to be successful. Companies traditionally seek pediatric expertise from pediatric pharmacologists, pharmacometricians and pediatric subspecialists.

One of the current foci of the pharmaceutical industry is rare diseases, many of which begin in childhood, such as cystic fibrosis. For this type of drug development, the need for extra pediatric legislation to enforce consideration of children in the development process is less obvious than the case for the pediatric requirements for antihypertensive drugs. In the U.S., drugs for orphan diseases are exempt from mandatory pediatric requirements (PREA). We investigated the additional requirements for cystic fibrosis drug development by the EU pediatric committee.

## 7. EU PIPs on Drugs Targeting Cystic Fibrosis

An overview of all CF PIPs published by the EMA through August, 2014, is described in Table 1. From a presentation at the CF workshop in 2012, it is known that three more CF PIPs were under discussion in 2012 and that four CF PIPs were withdrawn during the PIP negotiation [29]. We classified the drugs into two categories: drugs with a new therapeutic principle and, hence, a breakthrough potential and antibiotics. In this manuscript, we will focus on the drugs with breakthrough potential.

**Table 1:** Overview cystic fibrosis (CF) pediatric investigation plan (PIP) decisions published by the European Medicines Agency (EMA) until December, 2014.

Drug Name and PIP Number	Drug Characteristics
Amikacin (sulfate) EMEA-000525-PIP01-08-M03	Inhaled antibiotic: Pseudomonas aeruginosa lung infection/colonization in cystic fibrosis patients; nontuberculous mycobacterial lung infection
Ataluren EMEA-000115-PIP02-09-M01*	PTC (premature termination codon) suppressor: small molecule designed to make ribosomes ignore premature stop codons
Aztreonam EMEA-000827-PIP01-09-M02**	Inhaled antibiotic: synthetic monocyclic beta-lactam antibiotic
Colistimethate Sodium EMEA-000176-PIP01-07-M04	Inhaled, decades-old antibiotic, fell out of favor for nephrotoxicity; last-resort antibiotics against MDR bacteria
Ivacaftor EMEA-000335-PIP01-08-M09	Potentiator approved for patients with the G551D mutation; improves their chloride transport by inducing non-conventional gating
Levofloxacin EMEA-001211-PIP01-11-M01	Inhaled fluoroquinolone antibiotic
Lumacaftor/ivacaftor EMEA-001582-PIP01-13	Combination of ivacaftor and lumacaftor
Lumacaftor EMEA-001173-PIP01-11	Corrector molecule for CF patients with the F508del mutation in the CFTR, enhances the number of channels of the CFTR protein at the cell surface.
Mannitol EMEA-000436-PIP01-08	White, crystalline solid for CF and bronchiectasis; inhaled as dry powder; osmotically draws water to thin the CF mucus
Tiotropium bromide EMEA-000035-PIP01-07-M05	Long-acting anticholinergic bronchodilator used for COPD and CF
Tobramycin EMEA-000184-PIP01-08-M01	Inhaled aminoglycoside antibiotic

* PIP01 indicates that the original PIP was the basis of the final decision. PIP02 means that only the second PIP led to a positive opinion. M01 means that this PIP has already gone through one modification round; ** the end of the PIP number indicates the number of modifications. Ivacaftor had so far to go through for 9 modifications; tiotropium bromide through 5.

## 8. New Medications with Break-Through Potential

### 8.1. Ivacaftor

Ivacaftor is a corrector molecule, able to modify the cystic fibrosis transmembrane conductance regulator (CFTCR) in CF patients with the G551D mutation. Four to five percent of CF patients have the G551D mutation. Ivacaftor was registered in the USA in January, 2012, for treatment of children with CF from the age of six years on, based on two pivotal trials in children that showed clear improvement of lung function (see above), and later in 2012 also by the EMA in Europe [9,10,11,12].

Table 2 gives an overview of the PIP measures for ivacaftor in its current version [36]. In short:

- Development of an age-appropriate formulation for children below six years, and a toxicity study in juvenile rats.
- Nine clinical studies in infants, children and adolescents from birth to 17 years.

**Table 2:** PIP measures for ivacaftor (Kalydeco).

Ivacaftor (Kalydeco), (EMEA-000335-PIP01-08-M08) Decision: 30 October 2013	
Quality	Age-appropriate formulation for children below 6 years of age with acceptability, palatability and compatibility testing.
Pre-Clinical	2. An oral (gavage) toxicity and toxicokinetics study in juvenile rats, with recovery.
Clinical	3. A randomized, double-blind, placebo-controlled, parallel group study to evaluate the efficacy and safety of ivacaftor (VX-770) in adolescents 12 to less than 18 years old (and adults) with cystic fibrosis (CF) and the G551D mutation. 4. A randomized, double-blind, placebo-controlled, parallel-group study to evaluate the efficacy and safety of ivacaftor (VX-770) in children with CF and the G551D mutation aged 6 to less than 12 years. 5. A randomized, double-blind, placebo-controlled, multicenter study in patients with CF with at least 1 allele of an R117H-CFTR mutation, aged six years and older. 6. A randomized, double-blind, placebo-controlled, multicenter study in (adults) and children with CF with the ΔF508/ΔF508-CFTR mutation, aged 12 to less than 18 years. 7. Rollover long-term safety and efficacy study in (adults) and children with CF aged 6 years and older with at least one allele of G551D-CFTR mutation. 8. A randomized, double-blind, placebo-controlled, crossover study to evaluate the effect of ivacaftor (VX-770) on lung clearance index in subjects with cystic fibrosis with the G551D mutation and a FEV_1_ above 90% predicted, 6 years and older. 9. A 2-part open-label study to evaluate the safety, pharmacokinetics (PK) and pharmacodynamics (PD) of ivacaftor (VX-770) in subjects with cystic fibrosis and a CFTR gating mutation, aged 2 to less than 6 years. 10. An open-label study to evaluate the safety, PK and PD of ivacaftor (VX-770) in newborns, infants and toddlers less than 24 months of age with cystic fibrosis and a CFTR gating mutation. 11. A 2-part, randomized, double blind, placebo controlled, crossover study with an open-label period to evaluate the efficacy and safety of ivacaftor in patients with cystic fibrosis aged 6 years or older, who have a non-G551D CFTR gating mutation.

The “01” in the PIP number shows that this is the original PIP, *i.e*., the developing company has not received a negative opinion. PIP01-08 shows that it was submitted for the first time in 2008, and the M09 shows that this is the ninth modification of the PIP so far. Only the newest version of the PIP decision is published on the EMA website. As outlined above, the regular PIP procedure takes almost a year (3 + 7 + 1 months), while the modification procedure is limited to 3 + 2 + 1 months, in short 0.5 years. Therefore, the ivacaftor team has, so far, assuming the best case scenario, spent 5.5 years in negotiating modifications in the ivacaftor PIP.

What is the added benefit of 5½ years additional negotiation regarding the Ivacaftor PIP? When we started considering this paper, the ivacaftor PIP decision on the EMA website at modification was on its seventh iteration and contained as measures beyond those listed in Table 2 one more requirement: development of a film-coated tablet. Obviously, this requirement is now regarded as fulfilled by the EMA and was removed from the most updated PIP decision. As ivacaftor was developed for children ≥ 6 years from the beginning anyway, we do not see the need for a pediatric committee to ask for the development of a tablet.

We compared the PIP decision of 2014 with the ivacaftor studies posted on http://www.clinicaltrials.gov [37]. As of August, 2014, 41 planned, ongoing or concluded studies are listed, but with our focus on ivacaftor alone, we excluded studies from our analysis where the company Vertex investigates ivacaftor alongside other projects from its development pipeline or investigated ivacaftor in a completely different disease, e.g., COPD (chronic obstructive pulmonary disease). This left 20 studies listed in Table 3.

To allow easy cross-referencing between the PIP decision and the www.clinicaltrials.gov-listed studies, we added into Table 3 (http://www.clinicaltrials.gov-listed ivacaftor studies) the corresponding PIP measure number.

The “quality” requirement of an age-appropriate formulation for children < 6 years corresponds to the PIP Study 1. In order to run a trial in children < 6 years, tablets are not an appropriate formulation. Therefore, for a company planning trials in children < 6 years, the “quality” requirement is not an enlightening add-on, but an obvious requirement anyway.

Which additional information will the non-clinical study in juvenile rats yield? It will look at organ development in juvenile rats on the background that the drug might eventually be used for chronic treatment in young (2–6 years old) and very young (from birth to < 2 years of age) children. Once results from such clinical studies are available, the juvenile rat studies will give “additional information”. What will this information be worth? This drug is already licensed for children six years and older. The drug will likely not be toxic in five-year-olds. Irrespective of approval, if ivacaftor in infants and toddlers (after birth up to two years) shows a clinical benefit, physicians will prescribe it to those patients. For registration in infants and toddlers, the juvenile rat study will not help the go/no-go decision.

An analysis of the PDCO-requested clinical studies [36] shows that they appear to simply reflect the sponsor’s (Vertex) development program:

- A phase 2 proof-of-concept study in CF patients ≥ 6 years (PIP Study 8, NCT01262352). This study was performed in 2011, with the study results posted on clinicaltrials.gov and published by Davies, 2013.
- Two pivotal studies in children 6–11 year (PIP Study 3, NCT00909532) and in adolescents and adults ≥12 years old (PIP Study 4, NCT00909727) were the pivotal studies agreed upon by the FDA that were the core of the FDA registration in 2012.
- The two pivotal studies were supplemented by a roll-over study (PIP Study 7, NCT00953706) for those patients that had participated in one of the pivotal studies.
- There are three PIP-requested studies on patients with a mutation other than G551D (R117H-CFTR mutation, PIP Study 5, NCT01614457; ΔF508/ΔF508-CFTR mutation, PIP Study 6, NCT00953706; non-G551D CFTR gating mutation, PIP Study 11, NCT02070744 and NCT01614470.). These three studies (four on clinicaltrials.gov, as one is a two-part study) appear as part of the Vertex development plan to expand the range of indications for ivacaftor. The study on the ΔF508/ΔF508-CFTR mutation was negative and resulted in a limitation-of-use paragraph in the FDA ivacaftor prescription information. The study on the non-G551D CFTR gating mutation resulted in an extension of the prescription information to the CFTR mutations, G551D, G1244E, G1349D, G178R, G551S, S1251N, S1255P, S549N and S549R, that were entered into the U.S. ivacaftor prescription information and somewhat later also accepted by the EU. The study on the R117H-CFTR mutation was completed in October, 2013, with no results published yet. It can be concluded that all three studies on mutations other than G551D do not appear to originate from genuine pediatric PDCO requests, but were part of the original Vertex development plan.
- The two-part study in children aged 2–5 years (PIP Study 9, NCT01705145 and NCT01946412 ) is a routine expansion of the use of ivacaftor in children < 6 years of age.
- The study in newborns, infants and toddlers (PIP Study 10, not on clinicaltrials.gov) fits into the PDCO logic to request drug development for all age groups. At this stage, it not clear to what degree such a study makes sense. CF children today survive the first two years of life, and the therapeutic benefit of ivacaftor in this age group remains to be seen.
- Furthermore, the non-PIP-triggered additional clinical trials published on clinicaltrials.gov were analyzed. In Table 3, Studies 25, 26, 28, 30 and 39 obviously investigate challenges that have arisen during the ivacaftor clinical development and led to the sponsor’s decision to investigate in a systematic manner. Studies 1, 2, 38, 40 and 41 are university-sponsored trials typical for drugs that are already on the market. Academic researchers seek grants to investigate additional aspects of the existing drug.
- What was the potential benefit of the original PIP and nine additional modifications? PIP modifications become necessary when key elements in the sponsor’s study commitments cannot be maintained. The most frequent reason for this is delays due to recruitment challenges or other technical problems. In its 2011 report to the EU Commission, the EMA estimated the need for 3–5 modifications over the development time of a new drug [9]. Modifications must be requested by the sponsor.
- Evaluating the original registration of ivacaftor in the U.S. and the new registration of additional indications (*i.e.*, other CFTR mutations) by the FDA, it is apparent that in both cases, the EU lagged behind the U.S. by six months. It took a negotiation period of 5.5 years with PDCO to demonstrate studies that would have likely been performed without any negotiation. One example involves a PIP requirement for a tablet that must have been initially planned from the beginning, so this requirement is of minimal importance. The same holds true for the development of a small child-friendly formulation and for all clinical studies, with possibly the exceptions of PIP Study 10 and the juvenile rat study. The tentative conclusion is that the 5.5 years of continuous negotiation with the PDCO did not yield anything beyond what the sponsor had likely planned. Furthermore, Studies 1, 2, 25, 26, 28, 30, 38, 39, 40 and 41 were designed without PDCO involvement. Therefore, it is hard to assess if the sponsor’s 5.5 years of negotiation with the PDCO added beneficial aspects to the development of ivacaftor.

**Table 3:** Ivacaftor studies on http://www.clinicaltrials.gov.

Clinicaltrials.gov ID (Study No. in the List of All Ivacaftor Studies *)	Study Title	Age (Years)	Start/End	Sponsor/PIP Study No.
NCT02194881 [1]	Ivacaftor in French CF Patients with a G551D Mutation	≥6	August, 2014–May, 2015	Hôpitaux de Paris, France; NiP *
NCT01784419 [3]	Short-Term Effects of Ivacaftor in Non-G551D Cystic Fibrosis Patients	≥6	October, 2013–December, 2014	University of California, San Francisco; NiP
NCT01705145 [8]	Study of Ivacaftor in Cystic Fibrosis Subjects 2 through 5 Years of Age With a CTFR Gating Mutation	2–5	December, 2012–March, 2014	Vertex ≠ PIP 9
NCT01946412 [12]	Roll-Over Study of Ivacaftor in CF Pediatric Subjects with a CFTR Gating Mutation	≥2	December, 2013–November, 2015	Vertex ≠ PIP 9, Part 2
NCT01614457 [16]	Study of Ivacaftor in Subjects with Cystic Fibrosis Who Have the R117H-CFTR Mutation (KONDUCT)	≥6	June, 2012–October, 2013	Vertex ≠ PIP 5
NCT01707290 [17]	Rollover Study of Ivacaftor in Subjects with Cystic Fibrosis and a Non-G551D CFTR Mutation (KONTINUE)	≥6	February, 2013–June, 2016	Vertex ≠ PIP 11, Part 2
NCT01614470 [19]	Ivacaftor in Subjects with Cystic Fibrosis Who Have a Non-G551D CFTR Gating Mutation (KONNECTION)	≥6	July, 2012–October, 2013	Vertex ≠ PIP 11
NCT00909532 [21]	Ivacaftor in Cystic Fibrosis Subjects Aged 12 Years and Older with the G551D Mutation (STRIVE)	≥12	June, 2009–January, 2011	Vertex ≠ PIP 3
NCT00909727 [22]	Ivacaftor in Cystic Fibrosis Subjects Aged 6 to 11 Years with the G551D Mutation (ENVISION)	6–11	March, 2010–April, 2011	Vertex ≠ PIP 4
NCT00953706 [23]	Ivacaftor in CF Subjects Aged 12 Years and Older Homozygous for the F508del-CFTR Mutation (DISCOVER)	≥12	September, 2009–July, 2010	Vertex ≠ PIP 6
NCT01161537 [25]	Effect of VX-770 on Hyperpolarized Helium-3 Magnetic Resonance Imaging in Subjects With CF and the G551D Mutation	≥12	October, 2010–February, 2013	Vertex Not in PIP
NCT01685801 [26]	Pilot Study Testing the Effect of Ivacaftor on Lung Function in Subjects with Cystic Fibrosis and Residual CFTR Function	≥12	September, 2012–April, 2014	Vertex Not in PIP
NCT02039986 [28]	Ivacaftor (Kalydeco) and Insulin in Cystic Fibrosis (CF)	≥12	January, 2014–January, 2016	Children's Hospital of Philadelphia; NiP
NCT01863238 [30]	Ocular Safety Study of Ivacaftor-Treated Pediatric Patients 11 Years of Age or Younger with Cystic Fibrosis	6–11	May, 2013–June, 2016	Vertex Not in PIP
NCT00953706 [31]	Study of VX-770 in Cystic Fibrosis Subjects (PERSIST)	≥6	July, 2010–May, 2014	Vertex PIP 7
NCT01262352 [36]	Study of the Effect of Ivacaftor on Lung Clearance Index in Subjects with Cystic Fibrosis and the G551D Mutation	≥6	January, 2011–November, 2011	Vertex PIP 8
NCT02141464 [38]	Energy Balance and Weight Gain with Ivacaftor Treatment	≥6	March, 2014–April, 2015	Children's Hospital of Philadelphia; NiP
NCT01381289 [39]	VX-770 Expanded Access Program; obsolete since marketing approval	≥6	N/A	Vertex; NiP
NCT01549314 [40]	Cystic Fibrosis-Related Bone Disease: the Role of CFTR	6–75	April, 2012–December, 2015	Massachusetts Gen.l Hospital; NiP
NCT01951833 [41]	Long-Term Significance (Survival) of LCI (Lung Clearance Index) in Patients with Cystic Fibrosis	6–65	January, 2013–May, 2015	Univ Hospital St Luc, Brussels; NiP

*NiP = Not in the PIP. * Forty one ivacaftor studies on clinicaltrials.gov; only ivacaftor alone in CF was analyzed.

### 8.2. Lumacaftor

Lumacaftor is designed to help patients with the F508del mutation in CFTR. About 60% of CF patients are affected by this mutation. Lumacaftor is in late-stage clinical development. The two pivotal studies in patients six years and older showed statistically-significant improvement compared to the comparison groups. The sponsor, Vertex, plans to submit for registration in the U.S. towards the end of 2014.

Table 4 shows the PIP measures for lumacaftor. In short:

- Development of a tablet and of an age-appropriate formulation;
- Seven toxicity studies in juvenile and adult rats and dogs;
- Six clinical studies to investigate PK, safety and efficacy in infants, children and adolescents.

**Table 4:** PIP measures for lumacaftor.

Quality	1. Development of a film-coated tablet. 2. Development of an age-appropriate oral formulation for children below 6 years of age with testing of acceptability, palatability and compatibility of the formulation with common food and drinks.
Non-Clinical	3. Six-month oral (gavage) toxicity and toxicokinetics study with lumacaftor and VRT-0995096 in rats with a 1-month recovery (VX-809-TX-012). 4. Three-month oral (gavage) toxicity and toxicokinetics study with lumacaftor, ivacaftor and VRT-0995096 in rats with a 1-month recovery (VX-809-TX-013). 5. Twelve month oral (gavage) toxicity and toxicokinetics study with lumacaftor in beagle dogs with a 1-month recovery (VX-809-TX-014). 6. Developmental and perinatal/postnatal reproduction toxicity study in rats, including a postnatal behavioral/functional evaluation (VX-809-TX-017). 7. Fertility and early embryonic development study in rats with lumacaftor and VRT-0995096 (VX-809-TX-016). 8. Oral (gavage) dose range finding study in juvenile rats (VX-809-TX). 9. Oral (gavage) toxicity and toxicokinetics study in juvenile rats with 4-week recovery (VX-809-TX).
Clinical	10. Open-label, parallel-group, multicenter trial to evaluate the PK of lumacaftor, ivacaftor and their respective major circulating metabolites in patients with cystic fibrosis (CF) and a F508del-CFTR mutation aged 6 to less than 18 years (Study P1). 11. Randomized, double-blind, placebo-controlled trial to evaluate efficacy and safety in patients with CF and an F508del-CFTR mutation aged 6 to less than 12 years (Study P2a). 12. Randomized, double-blind, placebo-controlled trial to evaluate efficacy and safety in patients with CF and a F508del-CFTR mutation aged 12 to less than 18 years (and adults) (Study P2b). 13. Rollover open-label long-term safety and efficacy study in patients with CF and a F508del-CFTR mutation aged 6 to less than 18 years (and adults) (Study P3). 14. Pharmacokinetic and pharmacodynamic study in patients with CF and an F508del-CFTR mutation aged 0 to less than 6 years (P4). 15. Relative bioavailability study to characterize the PK of the pediatric age-appropriate formulation relative to the tablet formulation in healthy adults.

Lumacaftor is not yet registered. Essentially, the same considerations apply as to ivacaftor above.

To get a bit more into depth, we searched for clinical trials on the U.S. clinical trials registry [37]. There are 12 active trials at present, details are listed in Table 5.

Studies 2, 3, 6, 7 could correspond to PIP studies, but probably are identical to FDA-approved studies. Studies 1, 3, 4, 5, 8, 9, 10, 11, 12 are usual phase 1 and other early studies of the usual registration package. Obviously, the EMA PDCO-triggered PIP has so far not led to any additional recruitment into PDCO-triggered studies, which is to be seen as positive.

**Table 5:** Lumacaftor studies on http://www.clinicaltrials.gov.

1	Completed	A phase 1 study to examine the drug-drug interaction of ciprofloxacin, itraconazole and rifampin on the combination of lumacaftor with ivacaftor in healthy adult subjects
2	Active, not recruiting	Study of lumacaftor in combination with ivacaftor in CF subjects 6 to 11 years of age with the F508del-CFTR mutation
3	Recruiting	Phase 1, QT/QTC interval study in healthy subjects
4	Recruiting	Study of VX-809 alone and in combination with VX-770 in CF patients homozygous or heterozygous for the F508del-CFTR mutation
5	Completed	a phase 1 study to investigate the food effect of lumacaftor in combination with ivacaftor
6	Active, not recruiting	A study of lumacaftor in combination with ivacaftor in CF subjects aged 12 years and older who are homozygous for the F508del-CFTR mutation (TRAFFIC)
7	Active, not recruiting	A study of lumacaftor in combination with ivacaftor in CF subjects aged 12 years and older who are homozygous for the F508del-CFTR mutation (TRANSPORT)
8	Completed	Study of lumacaftor in combination with ivacaftor in subjects with moderate hepatic impairment and healthy subjects
9	Enrolling by invitation	A phase 3 rollover study of lumacaftor in combination with ivacaftor in subjects 12 years and older with cystic fibrosis
10	Active, not recruiting	Study of VX-809 in CF subjects with the ∆F508-CFTR gene mutation
11	Active, not recruiting	Drug-drug interaction study of VX-770 and VX-809 in healthy subjects
12	Active, not recruiting	Drug-drug interaction study of VX-809 and VX-770 in healthy subjects

### 8.3. Lumacaftor/Ivacaftor

In November 2014, Vertex submitted the combination of lumacaftor and ivacaftor for cystic fibrosis patients aged 12 years and older with two copies of the F508del mutation in the CFTR gene for registration both in the U.S. and in the EU. Table 6 lists the PIP measures for this combination: two quality measures (a film-coated tablet for children 6–11 years old and an age-appropriate formulation for children < 6 years; seven non-clinical studies in juvenile and adult animals of varies species; and eight clinical studies).

We expect that Vertex will again develop this combination in close cooperation with the FDA and will in parallel submit one PIP modification after the other. Eventually, once the combination has been approved by the FDA, the EMA CHMP will approve it, as well, and the PDCO will continue to discuss and decide further modifications.

**Table 6:** PIP measures for lumacaftor/ivacaftor.

Lumacaftor/ivacaftor (EMEA-001582-PIP01-13) 6 May 2014; completion until July 2017	
Indication: cystic fibrosis	
Quality	Development of an age-appropriate fixed-dose combination film-coated tablet for children aged 6 to less than 12 years old. Development of an age-appropriate oral solid formulation for children below 6 years of age.
Non-clinical	3. Six-month oral (gavage) toxicity and toxicokinetics study with lumacaftor and VRT-0995096 in rats with a 1-month recovery (VX-809-TX-012). 4. Six-month oral (gavage) toxicity and toxicokinetics study with lumacaftor, ivacaftor and VRT-0995096 in rats with a 1-month recovery (VX-809-TX-013). 5. Twelve-month oral (gavage) toxicity and toxicokinetics study with lumacaftor in beagle dogs with a 1-month recovery (VX-809-TX-014). 6. Developmental and perinatal/postnatal reproduction toxicity study in rats, including a postnatal behavioral/functional evaluation (VX-809-TX-017). 7. Fertility and early embryonic development study in rats with lumacaftor and VRT-0995096 (VX-809-TX-016). 8. Oral (gavage) dose range finding study in juvenile rats (VX-809-TX). 9. Oral (gavage) toxicity and toxicokinetics study in juvenile rats with 4-week recovery (VX-809-TX).
Clinical	10. Open-label, multi-dose study to evaluate the pharmacokinetics (PK) and safety of lumacaftor in combination with ivacaftor and their respective major circulating metabolites in subjects with cystic fibrosis (CF) who are homozygous for the F508del-CFTR mutation aged 6 to less than 12 years (Study VX13-809-011). 11. Randomized, double-blind, placebo-controlled study to evaluate efficacy and safety and PK in subjects with CF homozygous for the F508del-CFTR mutation aged 6 to less than 12 years. 12. Randomized, double-blind, placebo-controlled study to evaluate efficacy and safety in subjects with CF who are homozygous for the F508del-CFTR mutation aged 12 to less than 18 years (and adults) (Study VX12-809-103). 13. Randomized, double-blind, placebo-controlled study to evaluate efficacy and safety in subjects with CF who are homozygous for the F508del-CFTR mutation aged 12 to less than 18 years (and adults) (Study VX12-809-104). 14. Rollover open-label long-term safety and efficacy study in subjects with CF who are homozygous for the F508del-CFTR mutation aged 6 to less than 18 years (and adults) (Study VX12-809-105). 15. Pharmacokinetic and safety study in subjects with CF who are homozygous for the F508del-CFTR mutation aged 2 to less than 6 years. 16. Pharmacokinetic and safety study in subjects with CF who are homozygous for the F508del-CFTR mutation from birth to less than 2 years of age. 17. Relative bioavailability study to characterize the PK of the pediatric age-appropriate formulation relative to the tablet formulation in healthy adults.

### 8.4. Ataluren

Ataluren is a novel, small-molecular agent designed to make ribosomes ignore premature stop codons. It is currently in clinical development in two genetic disorders caused by nonsense mutations. Not in all, but in some people with cystic fibrosis and Duchenne muscular dystrophy, the disease is caused by nonsense mutations. Ataluren is in clinical development for indications for CF, Duchenne muscular dystrophy and other indications (www.clinicaltrials.gov). For CF, the first phase 3 study has been concluded and showed only a statistically not significant trend in favor of the ataluren treatment group [42,43].

Table 7 gives an overview of the “measures” that the developing company had to “propose” in order to get the PIP approved. It shows several features, in short:

- Development of an age-appropriate formulation to allow accurate dosing in children < 20 kg;
- Three pre-clinical toxicology studies in neonate beagle dogs;
- Five clinical studies, covering the areas pharmacodynamics (PD), pharmacokinetics (PK), safety and efficacy in patients between two and 17 years and PK/PD in patients between one and 23 months.

The five clinical studies include a dose finding study (Study 5), one pivotal phase 3 study (Study 6), one extension of the pivotal study (Study 7), one PK/PD study in children between two and six years old and one OK/PD study for children < 6 years old. This is a standard minimum program for a drug that aims in the first step to be registered in patients ≥ 6 years old, with an extension to the pivotal study plus two PK/PD studies in two younger age groups: 2 to < 6 years and one month to < 2 years.

**Table 7:** PIP measures for ataluren.

PIP Measures for Ataluren (EMEA-000115-PIP02-09) 4 May 2011; completion until December, 2016	
Indication: Treatment of cystic fibrosis due to nonsense mutation (nmCF);	
Patients concerned: Children from 28 days to less than 18 years of age;	
Formulation: Granules for oral suspension.	
Quality	Age-appropriate formulation and dosing device that allows accurate dosing for children weighing less than 20 kg.
Non-Clinical	2. Seven-day tolerability and pharmacokinetic study of ataluren in neonatal beagle dogs. 3. Twenty eight-day dose range finding juvenile toxicology and toxicokinetic study of ataluren in beagle dogs. 4. Three-month juvenile toxicology and toxicokinetic study of ataluren in beagle dogs with a 3-month recovery period.
Clinical	5. Open-label, randomized, dose-ranging, challenge-dechallenge-rechallenge evaluation of pharmacodynamic (PD) activity, safety and pharmacokinetics of ataluren. 6. Randomized, double-blind, placebo-controlled study to evaluate the efficacy of ataluren adult and pediatric nmCF patients, ≥6 years of age. 7. Open-label extension study to evaluate safety of ataluren in adult and pediatric nmCF patients, ≥6 years of age. 8. Open-label, multiple dose trial to evaluate the pharmacokinetics (PK), safety and pharmacodynamic (PD) effect of ataluren in pediatric patients with nmCF aged from 2 to less than 6 years. 9. Open-label, multiple dose trial to evaluate the pharmacokinetics (PK) and safety of ataluren in pediatric patients with nmCF aged from 28 days to less than 23 months.

## 9. Critical Appraisal of the Ivacaftor, Lumacaftor and Ataluren PIPs

There are several common elements in these PIPs. The compounds have breakthrough potential for the treatment of CF. While ivacaftor and lumacaftor had successful phase 3 studies that led to the registration of ivacaftor in 2012 and to the planned submission of lumacaftor in 2014, ataluren is lagging behind, as the first phase 3 trial failed to show a statistically-significant superiority over the comparator group (28). Furthermore, in November, 2014, Vertex submitted the dossier for the combination of lumacaftor and ivacaftor to both FDA and EMA.

What has been the role of the clinical part of the PIP decisions?

- Ivacaftor: Investigating http://www.clinicaltrials.gov, it appears that all clinical trials represent the drug developer’s development program. It was planned to expand the indication for the ivacaftor beyond patients with the G552D mutation (R117H-CFTR mutation, PIP Study 5, NCT01614457; ΔF508/ΔF508-CFTR mutation, PIP Study 6, NCT00953706; non-G551D CFTR gating mutation, PIP Study 11, NCT02070744 and NCT01614470.). These three studies (four on clinicaltrials.gov, as one is a two-part study) appear to be part of the Vertex development plan to expand the range of indications for ivacaftor. The study on the non-G551D CFTR gating mutation resulted in an extension of the prescription information to the CFTR mutations, G551D, G1244E, G1349D, G178R, G551S, S1251N, S1255P, S549N and S549R, which were entered into the U.S. ivacaftor prescription information and somewhat later also accepted by the EU. However, the study in infants and toddlers one month to < 2 years has not yet begun.
- Lumacaftor: No additional clinical requests beyond the developing company’s development plan, with the exception of the studies in children two to < 6 years old and one month to < 2 years old.
- Lumacaftor/ivacaftor: For the patients aged ≥ 12 years, the PIP decision contains the studies planned by the developer anyway, as the initial population target is patients ≥ 12 years of age. In addition, studies in patients aged 6–11, 2–6, 0–2 years, plus a bioavailability study for the pediatric age formulation are requested. There were no further comments beyond the characterization of the requests in the PIPs for ivacaftor and lumacaftor, respectively.
- Ataluren: No additional clinical requests beyond the developing company’s development plan, with the exception of the studies in children two to < 6 years old and one month to < 2 years old.

For the most advanced compound, ivacaftor, there are several studies, partially sponsored by the developing company and others sponsored by academia, which are running or in preparation, that are not part of the PIP requirements.

The PDCO has asked for studies in all age groups without consideration of the development stage of the respective drug. Ivacaftor is now registered, and clinical trials for children two to < 6 years old are underway, but not the requested trial for children one month to < 2 years old. Lumacaftor might be registered in 2015, and clinical trials in children < 6 years might then be prepared or not. Ataluren might or might not be registered one day in CF patients. The addition of the studies in young and very young patients has so far not had any influence on the actual drug development. This is one-size-fits-all approach should be modified, given the nature of the agent, the disease and clinical need.

So far, nine modifications have been discussed for the ivacaftor PIP, one for the lumacaftor PIP and one for the ataluren PIP. Looking at the development timelines (e.g., all ivacaftor measures are supposed to be fulfilled until December, 2016; see Table 2), it is obvious that planning, recruiting and reporting of the studies in very young patients will require several more modifications.

Regarding the non-clinical juvenile animal studies (one for ivacaftor, seven for lumacaftor, three for ataluren), it should be considered that the PDCO does not decide about drug registration. It is a body that has the power to block a drug’s registration at the EMA. The juvenile animal studies for all three PIPs can be justified to look for “additional safety signals”.

When work began on this paper more than a year ago, one clear consequence in the PIP requirements for ivacaftor was observed: the request to develop a tablet has been removed from Modification 9 in comparison to PIP Modification 7. This indicates that the PDCO is obviously satisfied that the company Vertex has by now developed a tablet.

## 10. Reflections

This analysis was undertaken to evaluate the additional benefits of the mandatory PIP discussions with the EU pediatric committee for three compounds with therapeutic breakthrough potential in the treatment of CF as an exploratory example. Regarding clinical trial development, the companies had previously planned the pediatric development in close interaction with the FDA. Ivacaftor was registered in the U.S. six months before the EU. After the registration of ivacaftor, the PIP discussions with PDCO continued, resulting in two more modifications thus far. Regarding the clinical requirements, this drug is critically important for those individuals with the appropriate mutation, and clearly, the drug will potentially help anyone with such a mutation and disease, irrespective of age. It is impossible to predict the number of PIP modifications until an ivacaftor clinical trial in infants and toddlers has been concluded, but the critical question remains: do the PIP requirements translate into a therapeutic benefit for children?

It is clear that for a drug used in children, a tablet, as well as a young child-friendly formulation will be required. This has clear benefits for the safety and treatment of the patient, as well as for the pharmaceutical company developing the drug. It would benefit the patients, regulators and pharmaceutical company to discuss this as part of an integrated process. For drugs primarily targeting pediatric diseases, the addition of a second negotiation round with the EMA pediatric committee in addition to the submission negotiation round to the CHMP does not automatically add safety or scientific advantages to straightforward drug development, but is resource consuming and costly.

## 11. Conclusions

This process has demonstrated that additional discussion rounds with the EMA PDCO have added additional time to a process that would likely have occurred without the additional five and one half years of negotiations for the clinical implementation of a breakthrough cystic fibrosis treatment. The company that developed ivacaftor was required to initially submit a PIP, followed by nine requests for modification, each taking six months of additional time, respectively, in total amounting to 5.5 years of negotiation. This amounted to the same approval as in the U.S., but took an additional six months. It would be desirable for EU pediatric legislation to allow safe, effective and novel compounds to the children that need them most in a timely manner.

We conclude that for new drugs that target exclusively or predominantly pediatric diseases, the current contribution of the EMA PDCO is less positive than intended, *i.e*., little to no benefit for the patients on one side and a seemingly endless bureaucratic procedure on the other side. We propose that in the planned review of the EU pediatric legislation, the involvement of PDCO is reconsidered to change the one-size-fits-all paradigm that all drugs initiate from adult indications and work their way into pediatrics. This change will reduce the time involved in the review of drugs that target exclusively or predominantly pediatric diseases, limit the numerous renegotiation steps and primarily allow a more rapid implementation of safe and effective therapeutics to treat pediatric diseases and improve the lives of our patients and their families.
